# Acupuncture at “Zusanli” (St.36) and “Sanyinjiao” (SP.6) Points on the Gastrointestinal Tract: A Study of the Bioavailability of ^99m^Tc-Sodium Pertechnetate in Rats

**DOI:** 10.1093/ecam/nep009

**Published:** 2010-10-18

**Authors:** Vasco Senna-Fernandes, Daisy L. M. França, Deise de Souza, Kelly C. M. Santos, Rafael S. Sousa, Cristiano V. Manoel, Sebastião D. Santos-Filho, Célia M. Cortez, Mario Bernardo-Filho, Marco Antonio M. Guimarães

**Affiliations:** ^1^Pós-Graduação de Ciências Médicas (PGCM), Universidade do Estado do Rio de Janeiro (UERJ), CEP: 20551-030, Rio de Janeiro, RJ, Brazil; ^2^Departamento de BiofÍsica e Biometria, Instituto de Biologia Roberto Alcântara Gomes (IBRAG), UERJ, Brazil; ^3^Academia Brasileira de Arte e Ciência Oriental/Colégio Brasileiro de Acupuntura (ABACO/CBA), Rio de Janeiro, Brazil; ^4^Departamento de Ciências Fisiológicas, UERJ, Brazil

## Abstract

The objective of this study is to investigate the differences of acupuncture effect between the Zusanli (St.36) and Sanyinjiao (SP.6) points on the gastrointestinal-tract (GIT) segment performed by the bioavailability of ^99m^Tc-sodium-pertechnetate (Na^99m^TcO_4_) in rats. Male Wistar rats (*n* = 21) were allocated into three groups of seven each. Group 1 was treated by acupuncture bilaterally at St.36; Group 2 at SP.6; and Group 3 was untreated (control). After 10 min of needle insertion in anesthetized rats, 0.3 mL of Na^99m^TcO_4_ (7.4 MBq) was injected via ocular-plexus. After 20 min, the exitus of animals was induced by cervical-dislocation and GIT organs isolated. However, immediately before the exitus procedure, blood was collected by cardiac-puncture for blood radio-labeling (BRL). The radioactivity uptake of the blood constituents was calculated together with the GIT organs by a well gamma counter. The percentage of injected dose per gram of tissue (%ID/g) of Na^99m^TcO_4_ was calculated for each GIT organs, while BRL was calculated in %ID. According to the one-way ANOVA, the stomach, jejunum, ileum from the treated groups (Group 1 and Group 2) had significant differences compared to the controls (Group 3). However, between the treated groups (Group 1 and Group 2), there were significant differences (*P* < .05) in the stomach, jejunum, ileum, cecum, transverse and rectum. In BRL analysis, Group 2 showed significant increase and decrease of the insoluble and soluble fractions of the blood cells, respectively (*P* < .0001). The authors suggest that St.36 may have a tendency of up-regulation effect on GIT, whereas SP.6, down-regulation effect. However, further rigorous experimental studies to examine the effectiveness of acupuncture in either acupuncture points need to be carried out.

## 1. Introduction

The gastrointestinal tract (GIT) syndromes related to the pain and functional disorders are common in the general population and may be treated successfully by acupuncture [1, 2]. This type of therapy is regarded as one of the most acceptable complementary therapy in Western world since it might be explained under the scientific view [3].

In traditional Chinese medicine (TCM), Zusanli (St.36) point of “The Stomach Meridian of Foot-Yangming" and Sanyinjiao (SP.6) point of “The Spleen Meridian of Foot-Taiyin" are commonly used in human acupuncture to treat a wide range of health conditions, including GIT disorders such as stomach ache, abdominal pain and distension, constipation, diarrhea, vomiting, dysentery, indigestion and others [4–6].

In animal research, St.36 and SP.6 are frequently used to study acupuncture effects on various physiological regulatory mechanisms and control systems changes, including gastrointestinal disorders, gastrointestinal motor dysfunction, visceral pain, gastric secretions (alter acid secretion), gastric and intestinal motilities, and regulation of brain-gut peptides [7–12]. In spite of several categories of assessment methods were applied to investigate the neuromodulation actions on the GIT, the explanation of the mechanisms and effects of acupuncture on those organs and tissues remains unclear [1, 2, 13]. Probably, it is not totally controlled by central nervous system (CNS) [14]. According to Kellow et al. [14] and Benarroch [15], all gastrointestinal reflexes that include ascending inhibitory reflexes (the rectocolonic and colonogastric reflexes), and descending excitatory reflexes (the gastrocolonic and ileocolonic reflexes) may also be controlled by the enteric nervous system (ENS). This system, regarded as the “Second Brain", provides capacity to induce GIT reflexes autonomously without any influence of the CNS.

In nuclear medicine, radionuclides such as ^99m^Tc are widely used to label molecular and cellular structures (radiopharmaceuticals or radiobiocomplexes). These labeled structures can be utilized to obtain scintigraphic images, or their bioavailability studies, which indicate the physiological processes including cells metabolism and circulation changes of a tissue [16].

The bioavailability of the radiobiocomplex ^99m^Tc-sodium pertechnetate (Na^99m^TcO_4_) is applied in clinical nuclear medicine to detect some GIT conditions such as intestinal hemorrhages, Merckel's diverticulum disease, and ectopic gastric tissues [16]. The unbound of pertechnetate anion (^99m^TcO_4_^−^) is distributed of throughout the vasculature and interstitial fluids by a slow diffusion mechanism, and it is concentrated in various organs including stomach and intestines [16]. However, the bioavailability of Na^99m^TcO_4_ can be changed in many circumstances by intrinsic factors; blood flow, capillary permeability, membrane transport, intracellular interactions, biotransformation and others. Extrinsic factors include the radiobiocomplex quality, patient's health status, diseases states, drugs (natural or synthetic type), dietary, invasive medical procedures and complementary therapies as acupuncture [17–19].

The aim of this study was to investigate the differences of acupuncture effects stimulated at the acupoints St.36 and SP.6 on the GIT-organs performed by the bioavailability of Na^99m^TcO_4_ in male Wistar rats.

## 2. Methods

### 2.1. Animals

The experimental design was approved by the Ethical Committee of the Instituto de Biologia Roberto Alcantara Gomes, Universidade do Estado do Rio de Janeiro, under the guidelines on the use of living animals in scientific investigations. Twenty-one healthy albino male Wistar rats, weighing 250–350 g at between 4 and 5 months of age were randomly allocated into three groups of seven; in Group 1 (*n* = 7), rats were treated with acupuncture at St.36 bilaterally; in Group 2 (*n* = 7), rats were treated with acupuncture at SP.6 bilaterally; and Group 3 (*n* = 7), rats were untreated (control group).

### 2.2. Experimental Design

The rats were fasted overnight with free access to water and anesthetized with an intraperitoneal injection of sodium thiopental 6.7% (*Thiopentax*) at the dose of 60 mg/kg/ip, body weight. Rats were placed in a ventral decubitus position, without immobilization in order to avoid animal stress and to eliminate emotional factors [19].

In Group 1, all rats were treated by manual acupuncture by inserting 5 mm deep at St.36, bilaterally, with stainless needle measuring 0.25 × 20 mm of length with guide-tube (*Sterile Acupuncture Needles Wujiang Shenli Medical and Health Material Co., Ltd, China*) [19]. In Group 2, animals received manual acupuncture by puncturing 5 mm deep into SP.6, bilaterally, with the same type of needle [18]. These acupoints in rat was positioned according to the palpation of the anatomical location of the cutaneous sites described by various authors in previous studies [10, 12, 18–21], and assured with an analogical paquimeter (*Sanny, Brazil*). The St.36 acupoint was located at between the tibia and fibula, laterally to the distal end of the cranial tuberosity of the tibia, *∼*5 mm lateral to the anterior tubercle of the tibia, in the tibialis anterior muscle, innervated by the deep peroneal nerve [12, 19, 20]. The SP.6 acupoint was situated approximately 3 mm proximal the largest prominence of the medial malleolus at the posterior tibia, and in the space between the Achilles tendon and the distal part of the tibia [10, 18, 21]. The animals from Group 3 were free from treatment.

### 2.3. Bioavailability of Radiobiocomplex Procedure

**Figure 1:**
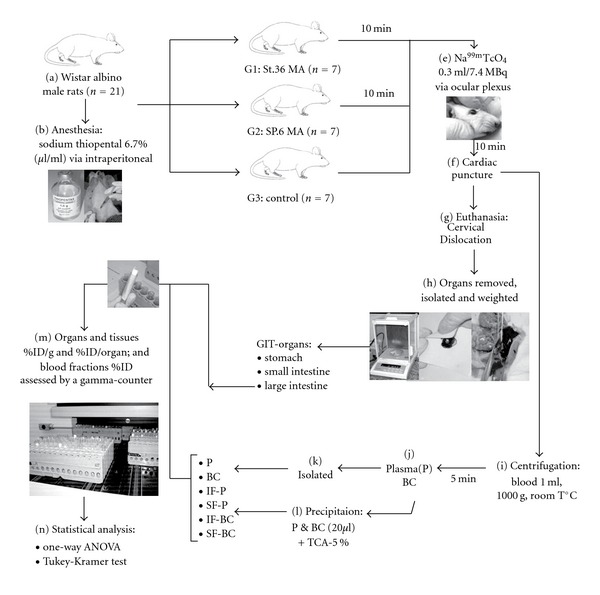
The flowgram of this study.

After 10 min of needle insertion, a dose of 0.3 mL of Na^99m^TcO_4_ (7.4 MBq) consisted of pertechnetate Tc-99m (^99m^TcO_4_^−^) recently eluted from a ^99^Mo/^99m^Tc column-type generator with the saline solution of 0.9% sodium chloride (Instituto de Pesquisas Energéticas e Nucleares, Comissão Nacional de Energia Nuclear, Brazil) was injected into the rat, via ocular plexus. The needles were withdrawn 20 min later, and the exitus of sedated animals was induced by cervical dislocation [19]. The selected GIT organs comprised of stomach, duodenum, jejunum, ileum, cecum, transverse colon and rectum of each animal [22] were identified and removed surgically as follows. The stomach was resected at the segment between right before the cardiac sphincter and after pylorus sphincter. In the small intestine segment, three samples of the rat have been isolated; (i) 20 mm of the duodenum after pylorus, (ii) 20 mm of the jejunum of the middle distance of the SI, (iii) 20 mm of the ileum before the ileocolic valve. In the large intestine, another three samples were resected too, (iv) the cecum (blinc sac), (v) 20 mm of the transverse colon and (vi) 20 mm of the rectum. All internal gut content including chyme, intestinal secretions and feces was gently removed to avoid false radioactivity uptake results. Then, all GIT organs were weighted by an analytic balance (Bioprecisa, Electronic Balance, FA2104N, São Paulo, Brazil) (Figure 1).

### 2.4. Blood Labeling Method

Before the exitus of rat, 2 mL of blood sample was withdrawn into heparinized tube (0.2 mL of heparin) via cardiac puncture, between the fifth and sixth intercostal space of the rat's left hemithorax. Then, 1 mL of blood was processed by a centrifugator (Bio-Eng 4000—Indústria e Comércio Ltd., São Paulo, Brat room temperature in 5 min. The plasma (P) and blood cells (BC) were isolated. Following samples (20 *μ*L) of P and BC were precipitated with 5% trichloroacetic acid whereas the soluble (S) and insoluble (I) fractions (F) of P and BC were determined. This procedure was used to assess the ability of the MA stimulation effects at St.36 and SP.6 on the blood constituents evaluated by blood labeling of Na^99m^TcO_4_ without using stannous chloride. Afterwards, the radioactivity of Na^99m^TcO_4_ uptake of all GIT organs and isolated blood constituents were measured by a gamma counter NaI (TI) (Cobra Auto-gamma, Packard Instrument Co.; Downers Grove, Illinois, USA). The percentage of injected dose of Na^99m^TcO_4_ per gram of tissue (%ID/g) of the GIT organs, were determined (Figure 1). The percentage of injected dose of Na^99m^TcO_4_ (%ID) of the blood constituents: P, BC, insoluble fractions of the plasma (IF-P) and blood cells (IF-BC), soluble fractions of the plasma (SF-P) and blood cells SF-BC was performed at the same time with the GIT organs samples [23] (Figure 1).

### 2.5. Statistical Analysis

For statistical analysis, the one-way ANOVA test, followed by the Tukey-Kramer Multiple Comparisons Test (TKMCT) were used in this study calculated the significant differences (*P* < .05). GraphPad InStat, version 3.01 and GraphPad Prism, version 3.00 (GraphPad Software Inc., San Diego Ca, USA) were applied to assess those tests.

## 3. Results

### 3.1. Bioavailability

**Figure 2:**
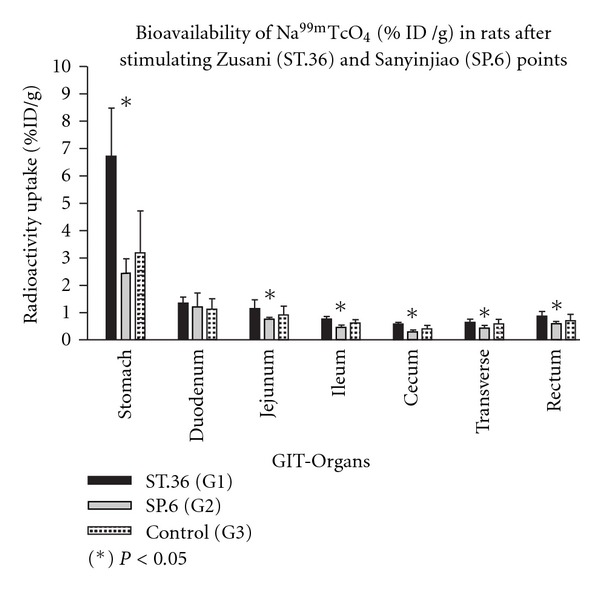
The distribution of the mean and standard deviation values of the uptake of Na^99m^TcO_4_ (%ID/g) in rats' gastrointestinal organs after stimulating Zusanli (St.36) and Sanyinjiao (SP.6) points. The organs and tissues of the stomach, jejunum, ileum, cecum, transverse colon and rectum were significantly different (*P* < .05) among the groups by one-way ANOVA followed by TKMCT; GIT—gastrointestinal tract. Asterisk indicates the result of the one-way ANOVA test.

Table 1 and Figure 2 show the statistical analyses carried out by the one-way ANOVA and TKMCT with significant differences of the radioactivity uptake in percentage of injected Na^99m^TcO_4_ dose per gram of organ (%ID/g) in GIT organs samples (stomach, jejunum, ileum, cecum, transverse colon, rectum) among three groups were significant (Table 1). According to one-way ANOVA, the stomach was the organ which had extremely significant increase of ^99m^TcO_4_^−^ uptake among the groups. According to the intergroup study with TKMCT, in between the treated and control groups it was observed that stimulating St.36 manually (Group 1) with needle in rat induced significant increase of uptake (%ID/g) in stomach and in jejunum compared to the control (Group 3). While stimulating SP.6 (Group 2) in the same way in rat provoked significant decrease of uptake in ileum compare to the control (Group 3). Moreover, in between the treated groups (Group 1 and Group 2), there were significant differences in the stomach, jejunum, ileum, cecum and rectum, considering *P* < .05. However, duodenum was not significant among the groups (Table 1 and Figure 2).

### 3.2. Blood Labeling

**Figure 3:**
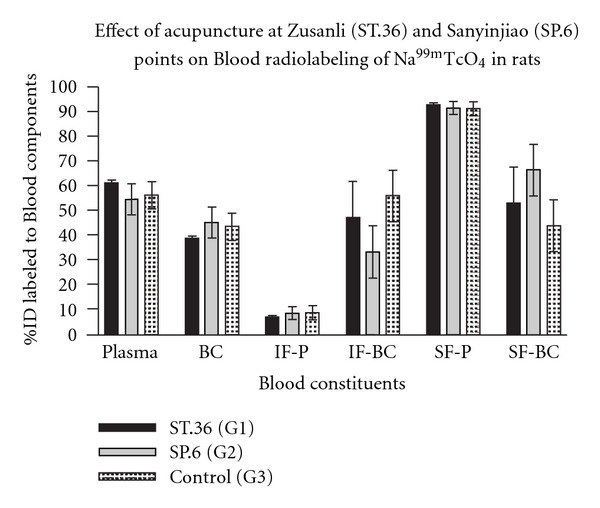
Effect of acupuncture at Zusanli (St.36) and Sanyinjiao (SP.6) points on the Blood radiolabeling of Na^99m^TcO_4_ in rats.

Table 2 and Figure 3 show the radiolabeling of blood constituents. IF-BC and SF-BC could induce significant differences. Stimulating SP.6 manually (Group 1) could induce significant decrease of IF-BC and increase of SF-BC compared to the control (Group 3).

## 4. Discussion

In Neuroscience, the somatovisceral reflex response, regarded as one of the neuromodulatory mechanisms of the autonomous nervous system, is suggested to be used to explain the actions of acupuncture through the relationship between acupoint and organ, empirically mentioned in the theory of *Zang-Fu*, in TCM [13, 19]. Cho et al. [24] pointed out that the acupuncture effects on the organs are generally mediated by CNS. Curiously, in our data (not included in this article) there were no significant difference of uptake of radioactivity in the brain among the groups (*P* > .05), which may let us speculate that CNS does not have full control on the body organs and tissues. Therefore, the relationship between the acupoints and *zang-fu* organs, described in TCM, may be explained by the ENS activities, since it can control directly the GIT organs responses without influences of CNS [5, 14, 15].

In this study, the authors did not use the “sham acupuncture" group as a useful “inactive" control, since the degree of inactivity with non-acupoints remains largely undetermined. Several authors suggested that the needling of non-acupoints could produce unpredictable physiological effects since the placement of the needle in any cutaneous location of the body surface would elicit biological responses (inflammatory reflex), and therefore could complicate the interpretation of the results in sham acupuncture [25–28]. Moreover, a supposed sham acupoint site may be confused to another acupoint which was not already discovered, or not including in this research. For instance, if a non-acupoint site is determined on 5 mm distal to St.36 in rat, it may correspond to another unexpected acupoint (Shangjuxu-St.37 or Tiaokou-St.38) which may induce specific radioactivity uptake.

In spite of the specificity of acupuncture is still questioned [29], in this research the actions of acupuncture at different acupoints in rat altered the bioavailability of Na^99m^TcO_4_ with significant differences of radioactivity uptake in some GIT organs [18, 19]. According to our data, the stomach, some parts of the small intestine (jejunum and ileum) and large intestine of Group 1 (St.36 acupuncture) showed significant increase of radioactivity uptake when compared with Group 2 (SP.6 acupuncture). We suggested that St.36 may have more capacity to promote activity than SP.6 in almost all segments of the GIT. When comparing the St.36-acupunture group (Group 1) with control (Group 3), we only observed that the uptake of radioactivity in the stomach and jejunum samples from Group 1 was significantly superior to Group 3. On the other hand, the data showed that there were lack of statistically significant differences between the treated and control groups (Group 1 versus Group 3 and Group 2 versus Group 3). However we could observe that all the mean values from Group 1 were higher than Group 3, whereas all from Group 2 were lower than Group 3. These results suggest that acupuncture stimuli at St.36 in rat may have an up-regulation tendency in various GIT organs, while at SP.6, a down-regulation tendency. These findings may be correlated to the excitatory and/or inhibitory responses or therapeutic potential actions promoted by these acupoints on different types of gastrointestinal functions or disorders referred in previous studies [2, 12, 30].

In acupuncture research on the specificity of the acupoints, imaging techniques such as single positron emission tomography, and functional magnetic resonance imaging have been used to investigate the sensorimotor pathways between the CNS and peripheral system of the GIT including the Brain-Gut Axis [31]. These techniques could produce anatomical and/or physiological imaging by mapping changes in brain activity in response to sensory (acupuncture) stimuli [32], However, using the bioavailability of radiobiocomplexes, such as Na^99m^TcO_4_, to assess acupuncture effect, the potential actions of acupoints might be “quantified" by radioactivity uptake in the targeted organs which is directly related to the changes of the activity of these organs' cells metabolism and blood circulation [19].

## 5. Conclusion

In the present study, St.36 and SP.6 under MA stimulation in rats showed that they may have potential therapeutic actions on some parts of GIT organs, which are thought to be useful not only to complementary-alternative therapies, but also to Western medicine. However, further rigorous experimental studies to examine their effectiveness in either acupuncture therapies need to be performed.

## Figures and Tables

**Table 1:** Bioavailability of Na^99m^TcO_4_ of the Gastrointestinal organs in %ID/g in rats.

GIT-organs	Mean ± SD (%ID/g)			ANOVA	TKMCT		
	St.36 (Group 1)	SP.6 (Group 2)	Control (Group 3)	*P*-value	Group 1 versus Group 2	*P*-value Group 1 versus Group 3	Group 2 versus Group 3
Stomach	5.32 ± 1.31	2.47 ± 0.44	2.57 ± 0.72	< .0001	***	***	ns
Duodenum	1.35 ± 0.21	1.12 ± 0.44	1.27 ± 0.21	.3250	ns	ns	ns
Jejunum	1.06 ± 0.35	0.69 ± 0.12	0.79 ± 0.16	.0052	**	∗	ns
Ileum	0.76 ± 0.10	0.49 ± 0.07	0.63 ± 0.11	.0001	***	ns	ns
Cecum	0.58 ± 0.07	0.29 ± 0.07	0.41 ± 0.13	.0060	**	ns	ns
Transverse	0.64 ± 0.12	0.43 ± 0.10	0.58 ± 0.17	.0394	*	ns	ns
Rectum	0.86 ± 0.19	0.58 ± 0.10	0.71 ± 0.22	.0416	*	ns	ns

The uptake of Na^99m^TcO_4_ (%ID/g) in gastrointestinal organs after stimulating Zusanli (St.36) and Sanyinjiao (SP.6) points showed that the stomach, duodenum, jejunum, ileum, cecum, transverse colon were significant (*P* < .05) among the groups by one way ANOVA followed by Tukey test. %ID/g: percentage of injected dose per gram of tissue of each organ. (ns) *P* > .05: not significant; *P < .05: significant; **P < .01: very significant; ***P < .001: extremely significant.

**Table 2:** Effect of acupuncture at Zusanli (ST.36) and Sanyinjiao (SP.6) points on the blood labeling of Na^99m^TcO_4_ in rats.

Blood constituents	Mean ± SD (%ID/g)			ANOVA *P-*value
	St.36 (Group 1)	SP.6 (Group 2)	Control (Group 3)	
Plasma	54.69 ± 6.31	56.38 ± 5.47	61.25 ± 1.14	ns
RBC	45.31 ± 6.31	43.62 ± 5.47	38.75 ± 1.14	ns
IF-P	8.56 ± 2.69	8.77 ± 2.83	7.19 ± 0.62	ns
IF-BC	47.07 ± 14.65	33.48 ± 10.4	56.14 ± 10.49	.0085
SF-P	91.44 ± 2.69	91.23 ± 2.83	92.81 ± 0.62	ns
SF-BC	52.93 ± 14.65	66.52 ± 10.49	43.86 ± 10.49	.0086

The uptake of Na^99m^TcO_4_ in the blood compartments after stimulating St.36 and SP.6 showed that insoluble and soluble fractions of the red blood cells were significant (*P* < .01) among the groups by one-way ANOVA followed by TKMCT. TKMCT: Tukey-Kramer multiple comparisons test.
